# Molecular characterization and immunopathological investigation of *Avian reticuloendotheliosis virus* in breeder flocks in Egypt

**DOI:** 10.1186/s12985-024-02525-5

**Published:** 2024-10-22

**Authors:** Eman Abd El-Menamm Shosha, Ali Mahmoud Zanaty, Marwa Mostafa Darwesh, Ahmed Fotouh

**Affiliations:** 1 Virology Department, Faculty of Veterinary Medicine, New Valley University, The New Valley Governorate, Egypt; 2 Gene Analysis Unit, Reference Laboratory for Quality Control On Poultry, Animal Health Institute, Agriculture Research Center (ARC), Giza, Egypt; 3 Pathology Department, Faculty of Veterinary Medicine, Benha University, Banha, Egypt; 4 Pathology and Clinical Pathology Department, Faculty of Veterinary Medicine, New Valley University, The New Valley Governorate, Egypt

**Keywords:** *Reticuloendotheliosis virus (*REV*)*, PCR, Sequencing, Histopathology, Immunohistochemistry

## Abstract

**Background:**

*Reticuloendotheliosis virus* (REV) is an oncogenic immunosuppressive retrovirus that infects different kinds of avian species; posing significant economic losses to the poultry industry worldwide.

**Methods:**

In Egypt, there is an unidentified disease associated with the runting-stunting syndrome with neoplasia, suspected to be REV, that has been continuously monitored in several breeder flocks. To diagnose and analyze REV by cell cultures, enzyme-linked immunosorbent assay (ELISA), histopathological investigation, the polymerase chain reaction (PCR) test, and sequencing analysis, 200 blood samples, and 50 tissue specimens were collected. The current study targets the occurrence and genetic characteristics of a viral neoplastic disease, resembling REV infection, circulating in breeder flocks from 2022 to 2023 in the Ismailia, El-Sharqia, and El-Dakahliya governorates.

**Result:**

Here, REV was isolated on chicken embryo fibroblast cell culture; exhibiting cell aggregation, rounding, and cell detachments. Collectively, only 70 serum samples were positive for anti‐REV antibodies with seroprevalence rates of 35% based on the ELISA test. The histopathological observation demonstrated lymphoreticular tumors in the liver, spleen, and other examined organs. The immunohistochemical staining method confirmed the REV-positive signals in all examined organs (liver, kidney, spleen, bursa, ovaries) except for the heart. The PCR assay of the *LTR* gene assessed 370 base pairs with only 5 positive samples with a percentage of 16.6%. Three positive samples were further sequenced and submitted to the Genbank under accession numbers (PP763709, PP763710, PP763711). Phylogenetic analysis of the REV-*LTR* gene showed that our three isolates (Sharquia-1-REV, Ismilia-2-REV, Mansoura-3-REV) are REV subtype III which predominantly circulated in breeders in Egypt. These three isolates are highest similar to American, Chinese, and Taiwanese REV reference strains, and other Egyptian strains with nucleotide identity percentages of 100%, 99%, and 99%; respectively, and on the amino acid identity level were with (99–100%), (98%, 99%), (99%, 100%); respectively.

**Conclusions:**

This study established that REV infection was extensively distributed in the breeders and became one of the causes of the clinical outbreaks of tumors, raising awareness of REV as the causative agent of avian oncogenic disease in Egypt.

**Supplementary Information:**

The online version contains supplementary material available at 10.1186/s12985-024-02525-5.

## Introduction

*Reticuloendotheliosis virus* (REV) is an enveloped, single-stranded, positive-sense RNA virus belonging to the genus *Gammaretrovirus* of the family *Retroviridae* [1]. REV genome is nearly 8.3 kb in size and encodes 3 major genes as the group-specific antigen (*gag*), polymerase (*pol*), and envelope (*env*) genes flanked by three identical non-coding long terminal repeats (*LTR*) regions. The *LTR* sequences of REV contain signals to initiate and terminate the genome transcription [2]. Despite only a single serotype of REV has been distinguished, REV has been categorized into three antigenic subtypes, including subtype I (170A), II spleen necrosis virus (SNV), and III chick syncytial virus (CSV) [3].

REV is an infectious neoplastic, immunosuppressive retrovirus infecting various kinds of avian species causing a significant threat to the poultry industry worldwide. REV-T is a defective prototype strain that carries an oncogene capable of acute oncogenicity [4]. Whereas, other non-defective REV strains have been reported in different poultry species such as ducks, chickens, geese, turkeys, peafowls, pigeons, and pheasants [5, 6]. These representative REV non-defective strains are associated with a variety of disease manifestations, involving anemia, proventriculitis, immunosuppression, lymphoid neoplasia, acute reticulum cell neoplasia, and runting–stunting syndrome [1]. REV also causes other neoplastic manifestations such as myxosarcomas, fibrosarcomas, and renal adenocarcinomas [1].

REV tropism was noticed in kidneys, lymphoid organs, blood cells, and epithelial cells. REV is transmitted either horizontally by close contact with infected cases, contaminated vaccines, or insects, and vertically [7]. Currently, there is no vaccination strategy or specific medications available for REV. Thus, in commercial poultry farms, REV can be controlled generally by strict biosecurity programs, management approaches, and elimination of infected breeders [1, 8].

It is noteworthy to state that REV proviral DNA integration of the partial or total genome into other host cell genomes or it can be embedded within some avian DNA viruses such as Fowlpox virus (FWPV) [9], and Marek’s disease virus (MDV) [10, 11]. These mutagenesis consequences can potentially alter the viral biological functions including field or vaccinal strains resulting in reducing the vaccine efficacy [2, 12]. Additionally, using of contaminated avian vaccines resulted in serious immunosuppression in the poultry flocks [12]. Furthermore, REV infection is a potential contamination hazard that can persevere over a period of various years at the poultry production site [9]. Recently, co-infection of REV with other avian oncogenic viruses has been frequently reported in chicken flocks, contributing to the intensified disease severity, virus transmissibility, and great harm to the poultry industry [8, 13].

An additional concern is that the conventional tests applied for the differential diagnosis of lymphoid neoplastic infections are commonly viral isolation, ELISA, as well as histopathological examinations. Particularly, molecular and immunohistochemical diagnostic methods are often used to overcome the technical difficulties of these classical methods [14]. The PCR test besides sequencing analysis are sensitive, rapid, and precise approaches for diagnosis of REV infections [15].

Nonetheless, few previous studies have reported the presence of REV either solely or in coinfection with MDV, and FWPV [16, 17]. This retrospective study aimed to investigate and characterize REV isolates that are circulating in broiler breeders in El-Sharqia, Ismailia, and El-Dakahliya governorates, Egypt through serological assay, PCR technique, pathological observation, immunohistochemical method, and sequencing approaches.

## Materials and methods

### Clinical samples and background

In the production period, a total number of 20 broilers breeders’, aged from 23 to 61 weeks’ old were investigated from January 2022 to November 2023. The size of examined breeder farms was situated in three districts in Ismailia, El-Sharqia, and El-Dakahliya Governorates, Egypt (Table 1). 200 anticoagulant-treated blood samples were gathered from suspected diseased breeders for commercial ELISA technique. In addition, fifty tumor tissue specimens including liver, spleen, kidney, bursa, heart, and ovaries were obtained then removed aseptically and separated into 2 parts, the first portion was kept in 10% buffered formalin for histopathological investigation, while, the other portion was kept frozen at − 80 °C for subsequent molecular examinations. The clinically diseased chickens had shown, anorexia, runting, stunted growth, head swelling, lameness, and aberrant feathering with pathological findings involving markedly emaciated or stunted carcasses, prominent sternums, and enlargements in visceral organs mostly observed in liver and spleen. Also, miliary nodules in the liver, and spleen tissues. Importantly, several kidneys were hemorrhagic and enlarged with grayish-white nodular infiltrations.

**Table 1:** The detailed data of the examined breeders for REV investigation

Farm locality	Breeder farms	Farm capacity	Tissue specimens							Collection date
Ek-Sharqia	10	3000–15,000	Blood	Liver	Spleen	Kidney	Bursa	Heart	Ovaries	2022–2023
			100	8	5	4	1	3	2	
Ismailia	3		30	2	1	2	0	1	2	
El-Dakahliya	7		70	6	5	3	0	2	3	

### Immunopathological investigation

The collected tissue samples including liver, spleen, heart, kidney, bursa, and ovaries were fixed in 10% neutral buffered formalin, washed, then dehydrated, and finally embedded in paraffin. Furthermore, paraffin blocks were sectioned at a thickness of 5 µm, and subsequently stained using Giemsa stain and hematoxylin and eosin (H&E) for microscopical analysis [18]. Concerning Immunohistochemical staining, tissue specimens were fixed in 10% buffered neutral formalin, paraffin-embedded, sectioned at 4 µm thickness, and mounted on poly-l-lysine-coated slides. A commercial polyclonal antiserum of Reticuloendotheliosis (Charles River Laboratories, Wilmington, Massachusetts, USA) was utilized as a primary antibody in a concentration of (1:5000) in phosphate buffer saline (PBS), and then incubated overnight at 4 °C. Moreover, the secondary biotinylated anti-chicken antiserum (Vector) was applied in a concentration of 1:5000 in PBS, after that the slides were incubated at 37 °C for 1 h in a humid chamber. Additionally, the obtained tissue sections were also stained with a routine streptavidin–biotin/horseradish peroxidase (HRP)-conjugated immunohistochemical technique. The sections were stained regularly to be assessed microscopically with light microscopy following the approach mentioned by [19].

### REV isolation and serological assay

REV was isolated on the chicken embryo fibroblast cell line (CEF) that was obtained from VACSERA (Vaccine and Serum Association), Giza, Egypt. After sample processing, they were inoculated successfully in CEF tissue culture and then monitored continuously to confirm the virus growth. After that, the inoculated cells were incubated at 37 °C in a 5% CO_2_ incubator for five days per passage. Uninfected CEF cells acted as a negative control. The tissue cultures are monitored daily by accurately recording any cytopathic effects (CPE) following the protocol of [20, 21]. Consequently, after three times serial passages, the culture supernatants including REV proviral DNA were harvested to be confirmed by PCR analysis using primers specific for *LTR* genes. Concerning serological assay, the collected blood specimens were centrifuged at 3000 rpm for 15 min, then kept at – 20 °C until used for the subsequent antibody detection of REV by a commercial REV antibody ELISA test (ELISA Test Kit, IDEXX Laboratories, Inc., USA). Finally, the ELISA technique was carried out following the manufacturer’s specifications. Additionally, samples with an S/P ratio of antibody titers greater than 0.5 were considered positive.

### PCR method, partial sequencing, and phylogenetic analysis

Interestingly, PCR amplification test using Phusion® High-Fidelity DNA Polymerase (Thermo, MA, USA) used for REV-DNA detection following the manufacturer’s recommendations. The genomic DNA extraction was performed using the QIAamp MinElut Virus Spin Extraction kit (Qiagen, GmbH, Hilden, Germany) according to the manufacturer’s instructions. The pair of oligonucleotide primers supplies a PCR amplicon product size in 370 base pairs (bp) (Table 2) [22].

**Table 2:** Sequence of oligonucleotide primers, and expected PCR product sizes exclusively amplify of REV LTR gene

Primers	Sequence (5′–3′) direction	Reference
REV 5′ LTR Forward	5′-ACCTATGCCTCTTATTCCAC-3′	[22]
REV 5′ LTR Reverse	5′-CTGATGCTTGCCTTCAAC-3′	

Concerning sequencing, the QIAquick Gel Purification Kit (Qiagen, Hilden, Germany) used to purify our three positive amplicons, then subsequently sequenced using ABI PRISM BigDye Terminator v3.1 Cycle Sequencing Kit (Applied Biosystems, California, USA) via sets of primers corresponding to the *LTR* gene of REV. Moreover, the oligonucleotide sequence was detected by using ABI PRISM 3500 xl Genetic Analyzer (Life Technologies, California, USA). The available nucleotide and inferred amino acid REV sequences were directly aligned with other correlated reference REV strains retrieved from Genbank database using the Clustal W program. A phylogenetic tree was constructed using the MEGA-X program with the model of maximum likelihood method [23] and BioEdit software, with levels estimated using 1,000 bootstrap replicates [24].

## Results

### Clinical and gross findings

**Fig. 1:**
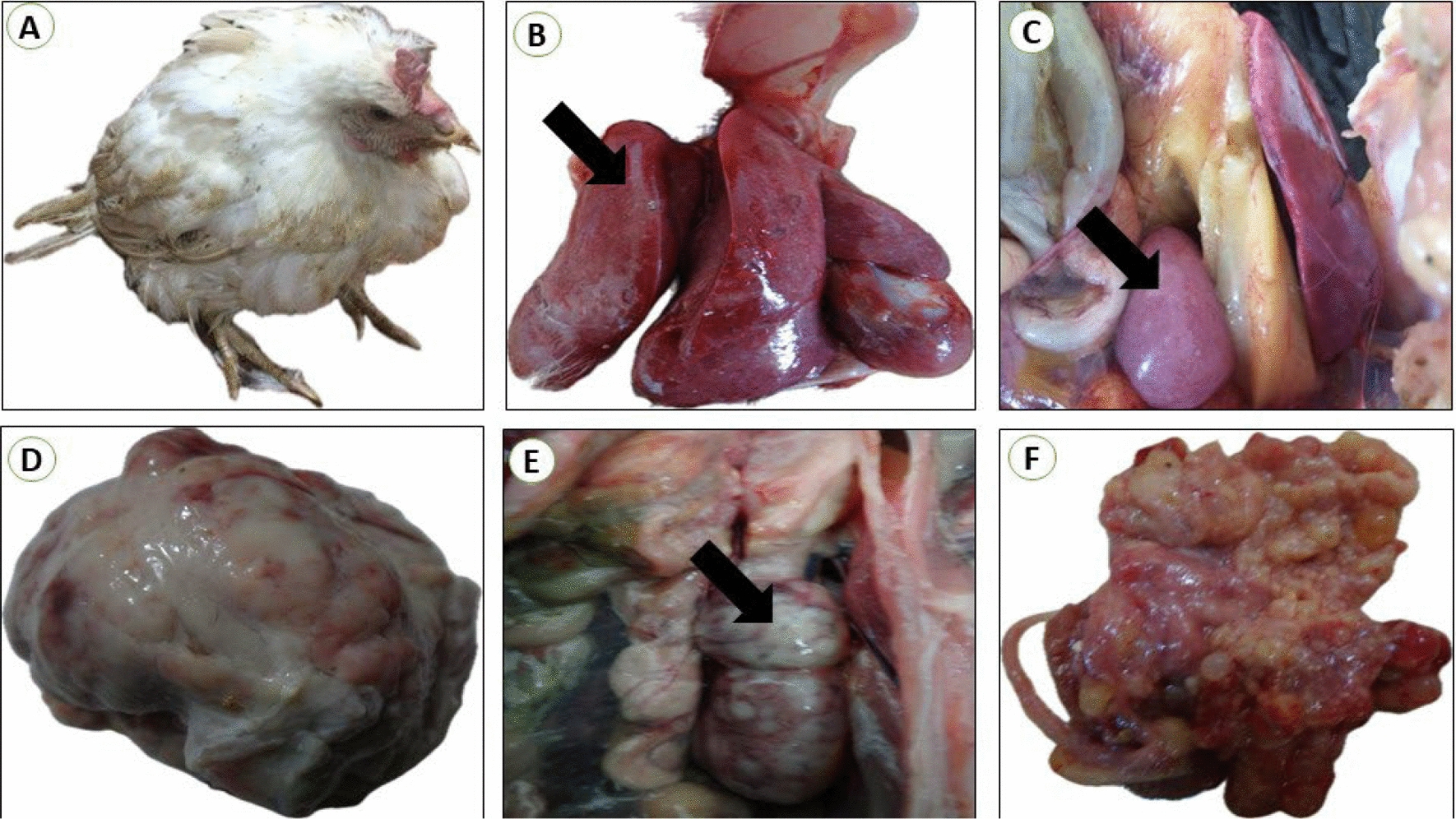
**A** Breeder chicken (36 weeks old) naturally infected with REV; showing severe emaciation with pale combs. **B** liver from breeder chickens (27 weeks old) naturally infected with REV; showing diffuse enlargement with numerous whitish uniformly nodules (1mm in diameter) scattered all over the surface (arrow). **C** Spleen from breeder chickens (23 weeks old) naturally infected with REV; showing diffuse enlargement with numerous whitish uniformly nodules (arrow). **D** Bursa from breeder chickens (25 weeks old) naturally infected with REV; showing diffuse enlargement with a hemorrhagic corrugated surface. **E** kidneys from breeder chickens (61 weeks old) naturally infected with REV; showing diffuse enlargement with mottling of surface (arrow). **F** Ovary from breeder chickens (49 weeks old) naturally infected with REV; showing undeveloped ova

Regarding positive farms, REV was detected in only 7 farms out of 20 with a seroprevalence rate of 35% with clinical symptoms as depression, emaciation, pale combs and wattles (Fig. 1A), pallor of the face, anorexia, runting-stunting symptom, unusual feathering, weakness, and lameness. The mortality rate was reported at 1.5–5%, whereas the morbidity rate was approximately 3%. Postmortem findings of dead birds mostly revealed moderately to markedly emaciated or stunted carcasses, prominent sternums, and enlargements in visceral organs mostly observed in the liver and spleen. The hepatomegaly may reach up to 3–5 of its normal size; also the diffuse enlargement of the liver occupies the whole abdomen. Besides, some liver tissues showed miliary nodules about 1–2 mm scattered all over their surface. (Fig. 1B). Likewise, the similar lesions were also recorded on the spleen (Fig. 1C). Occasionally, there is severe enlargement was demonstrated in the examined bursa (about 10 times more than the normal bursa) (Fig. 1D). Importantly, several kidneys were hemorrhagic and enlarged with grayish white nodules infiltrations (3–5 mm) (Fig. 1E). In ovaries, nonfunctional ovaries and regressed ovarian follicles were visible and detectable (Fig. 1F).

### Pathological and immunohistochemical examination

**Fig. 2:**
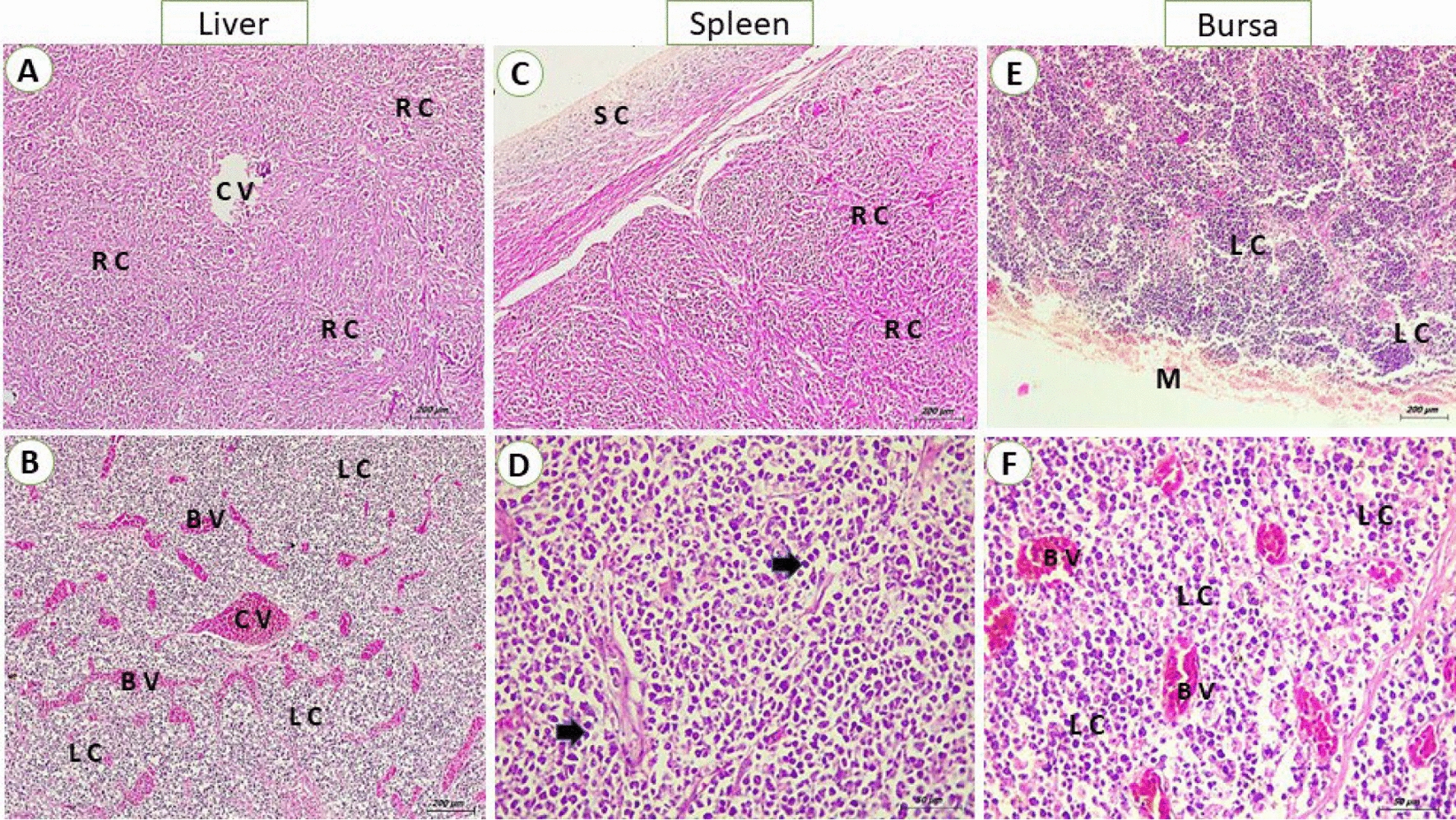
**A** liver from breeder chicken (38 weeks old) naturally infected with REV; showing massive primitive reticular cell proliferation (RC) around central vein (CV) (H&E, scale bar: 200 μm) **B** liver from breeder chicken (27 weeks old) naturally infected with REV; showing diffuse lymphoblastic cell infiltration (LC) with severely congested blood vessels (BV) (H&E, scale bar: 200 μm). **C** Spleen from breeder chicken (23 weeks old) naturally infected with REV; showing massive primitive reticular cell proliferation (RC) with thickening of the splenic capsule (SC) (H&E, scale bar: 200 μm). **D** Spleen from breeder chicken (35 weeks old) naturally infected with REV; showing diffuse lymphoblastic cell infiltration, the cells were hyperchromatic and pleomorphic (arrows) (H&E, scale bar: 50 μm). **E** Bursa from breeder chicken (61 weeks old) naturally infected with REV; showing diffuse lymphoblastic cell infiltration (LC) with severely degenerated mucosa (M (H&E, scale bar: 200 μm) **F** Bursa from breeder chicken (61 weeks old) naturally infected with REV; showing congestion of stroma blood vessels (BV) and lymphoblastic cell infiltration (LC) (H&E, scale bar: 50 μm)

**Fig. 3:**
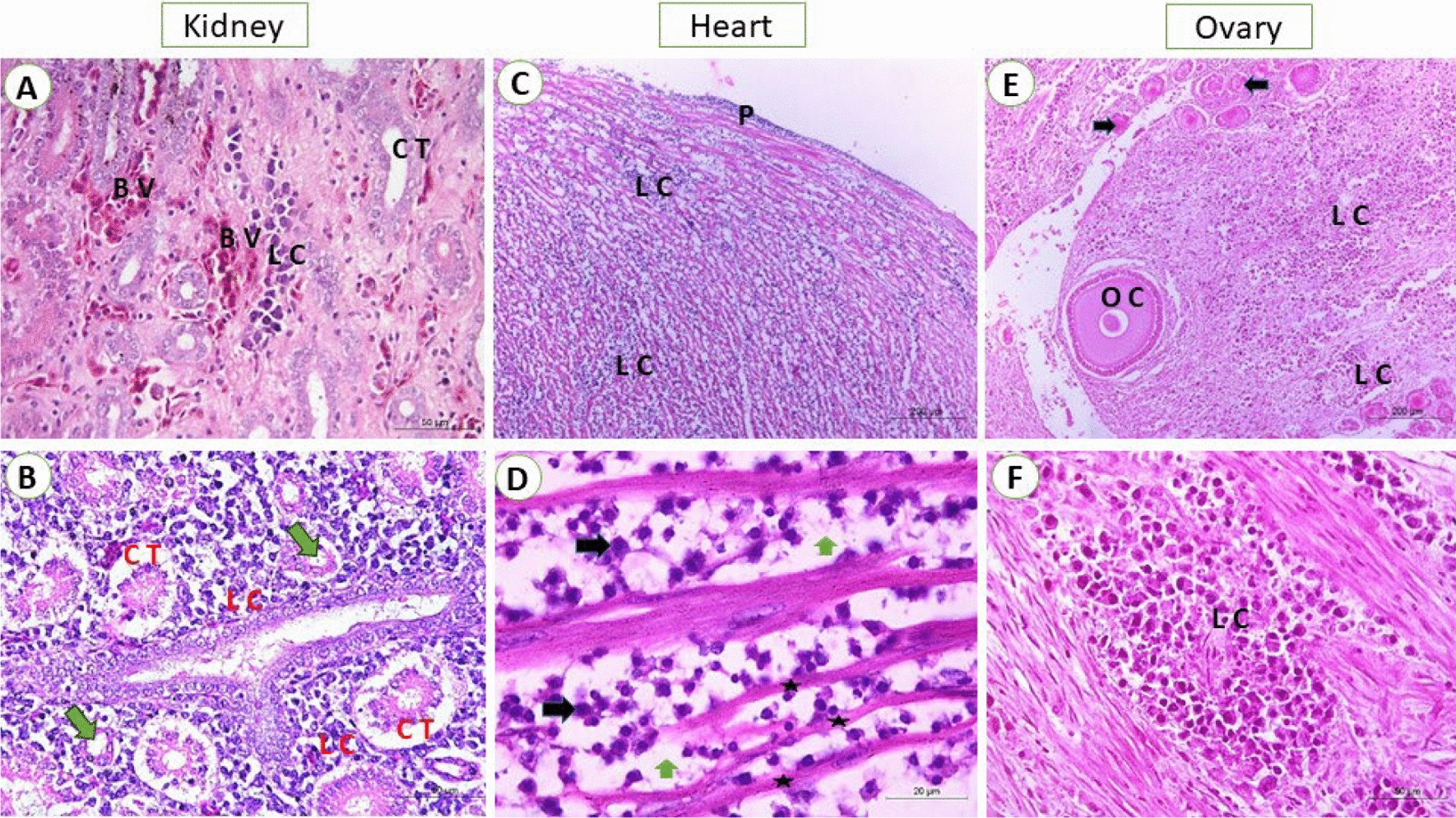
**A** kidney from breeder chicken (48 weeks old) naturally infected with REV; showing congestion and hemorrhage of interstitial tissue blood vessels (BV) with focal lymphoblastic cell infiltration (LC) (H&E, scale bar: 50 μm). **B** Kidney from breeder chicken (31 weeks old) naturally infected with REV; showing diffuse lymphoblastic cell infiltration (LC) with degenerated renal tubules (CT) and some tubules suffer from pressure atrophy (arrows) (H&E, scale bar: 50 μm). **C** Heart from breeder chicken (26 weeks old) naturally infected with REV; showing massive lymphocytic infiltration within myocardial fibers (LC) and under pericardium (P) (H&E, scale bar: 200 μm). **D** Higher magnification of the previous photo, lymphocytic cells showing pleomorphism and hyperchromasia (black arrow). Thinning and atrophy of myocardial fibers (astracs) with an increase of interstitial spaces (green arrows) (H&E, scale bar: 20 μm). **E** Ovary from breeder chicken (56 weeks old) naturally infected with REV; showing diffuse lymphoblastic cell infiltration (LC) with atrophied ovarian follicle (arrows) (H&E, scale bar: 200 μm). **F** Ovary from breeder chicken (25 weeks old) naturally infected with REV; showing focal lymphoblastic cell infiltration (LC) of ovarian stroma, the neoplastic cells show pleomorphism and atypism (H&E, scale bar: 50 μm)

**Fig. 4:**
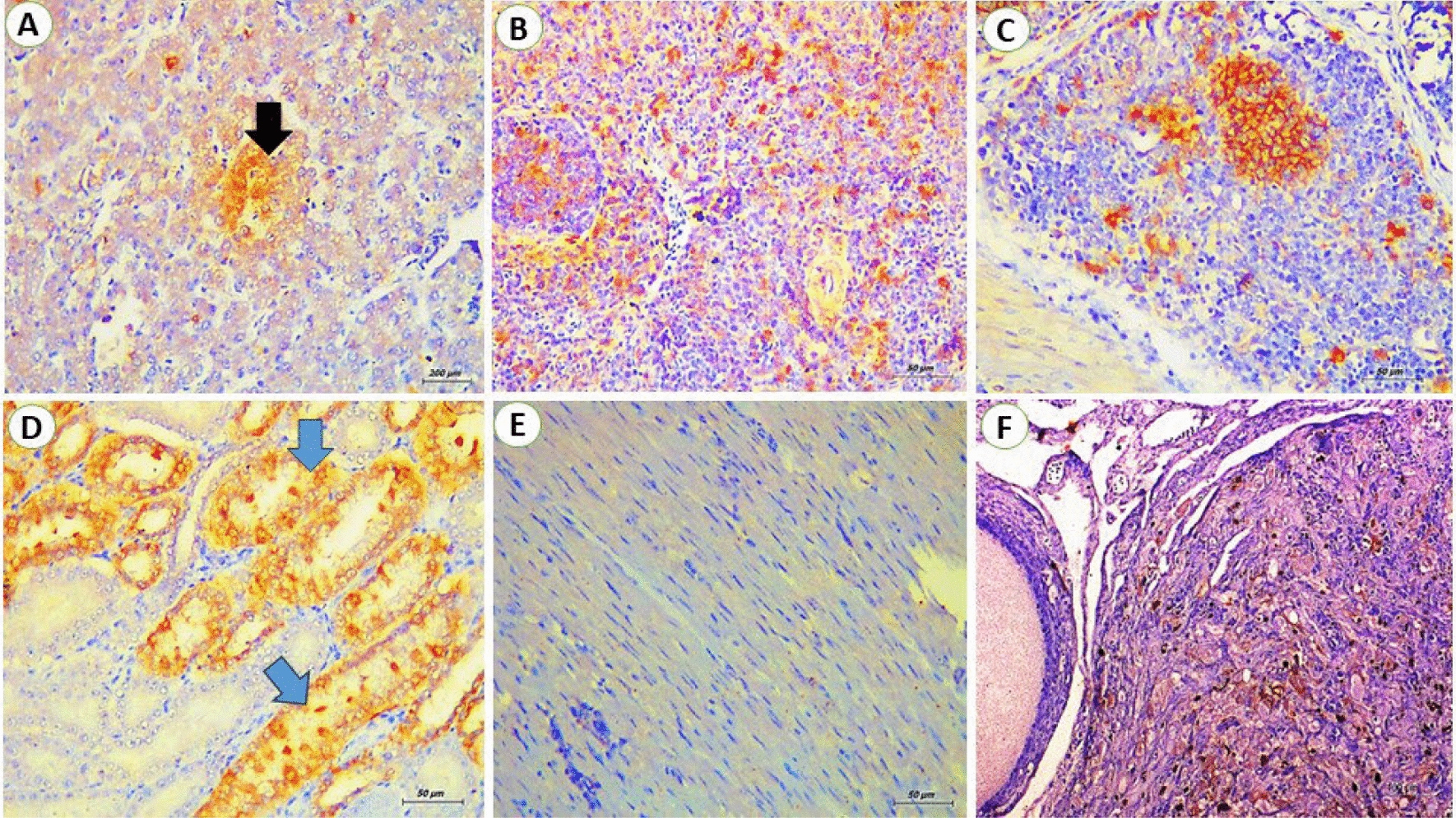
**A** liver; the brown granules indicated the positive reaction for the presence of virus particles in infiltrated lymphoblasts (arrow) within hepatic parenchyma (IHC stain, scale bar: 200 μm). **B** Spleen; the brown granules indicated the positive reaction for the presence of virus particles in infiltrated lymphoblasts in red and white pulps (IHC stain, scale bar: 50 μm). **C** Bursa; the brown granules indicated the positive reaction for the presence of virus particles in infiltrated lymphoblasts in lymphoid follicles (IHC stain, scale bar: 50 μm). **D** Kidney; the brown granules indicated the positive reaction for the presence of virus particles in the lining epithelium of renal tubules (arrows) (IHC stain, scale bar: 50 μm). **E** Heart; there is no positive reaction detected (IHC stain, scale bar: 50 μm). **F** Ovary; the brown granules indicated the positive reaction for the presence of virus particles in lymphocytes aggregated in ovarian stroma (IHC stain, scale bar: 50 μm)

In liver, reticular cell tumor was detected as a proliferation of primitive reticular cells that appear stellate, elliptical, and fusiform or spindle in shape. The reticular cells were observed either in focal areas or completely replaced the hepatocytes with activation of kupffer cells (Fig. 2A). The miliary form showed numerous numbers of small-size foci of aggregated lymphoblastic cells. In some cases, massive lymphoblastic cell infiltration replaces most of the hepatic parenchyma (Fig. 2B). In addition, the lymphoblastic cells are large mononuclear cells with poorly defined cell membranes. The nuclei were vesicular as there was margination and clumping of chromatin with the appearance of one or more noticeable acidophilic nucleoli. Also in spleen, the proliferation of reticular cells appeared as stellate, elliptical, or spindle shape and the nucleus took the shape of the cell with an interlacing of eosinophilic protoplasmic processes (Fig. 2C). The proliferated lymphocytes in white pulp showed pleomorphism and mitotic activity (Fig. 2D). Moreover, the diffuse proliferation of large lymphoid cells all over splenic tissue especially red pulp was seen in some cases. In addition, the bursa exhibited a diffuse proliferation of lymphoblast cells that infiltrate both congested interfollicular and stromal connective tissue as well as covering epithelium were observed. The lymphoblasts show pleomorphism, hyperchromasia, and mitotic figures (Fig. 2E–F). Meanwhile, the kidneys displayed diffuse lymphoblastic infiltrations all over the renal tissue. Furthermore, the renal tubules showed vacuolar degeneration, pressure atrophy, and sloughing of epithelial lining in some renal tubules together with pyknosis and karyorrhexis in some nuclei. Also, interstitial congestion and hemorrhage were prominent (Fig. 3A–B). Concerning heart, congestion of the myocardium was evident with interstitial edema. Vacuolation and hyalinization of the vascular wall were marked. Also, lymphocytic cells heavily infiltrate the myocardium so the myocardial fibers become thin and atrophied. The neoplastic cells showed pleomorphism, hyperchromatic, and mitotic figures (Fig. 3C–D). Subsequently, the ovarian stroma showed a massive infiltration of lymphocytic cells with congestion of vasculature. In some birds, the infiltrating neoplastic cells in the interfollicular spaces and around ovarian follicles cause atrophied or undeveloped ovarian follicles. Also, atrophy and degeneration of corpus oophorous which is surrounded by mononuclear pleomorphic lymphoid cells were observed (Fig. 3E–F). Regarding mmunohistochemistry, the liver showed intense brown granules staining of lymphoblastic cells. For the spleen, the presence of specific viral particles in lymphoblastic cells is distributed in red and white pulps. Indeed, the reaction in bursa was intense in the lymphoid follicles. Also, the lining epithelium of renal tubules of kidneys showed intense brown granules giving a positive reaction to the presence of viral particles. While, the myocardial tissues didn’t show any positive reaction. On screening of the ovary, the intense reaction was detected in lymphocytes aggregated in the ovarian stroma and ovarian follicle (Fig. 4A–F).

### CEF cell line and ELISA findings

Specifically, REV CPE was noticed subsequently after 72 h in inoculated CEF tissue culture in the form of cell aggregation, rounding, degeneration as well as massive cell detachments on the 5th day post-inoculation (d.p.i). In the ELISA results, serum samples revealed that only 70 samples were positive for anti‐REV antibodies with seroprevalence rates of 35% and the S/P ratio mean was 0.96 for sera of sick breeders. Whereas, 130 samples were negative for the REV antibody (75%) with S/P ratio mean of 0.35.

### REV detection and Proviral DNA sequencing

**Fig. 5:**
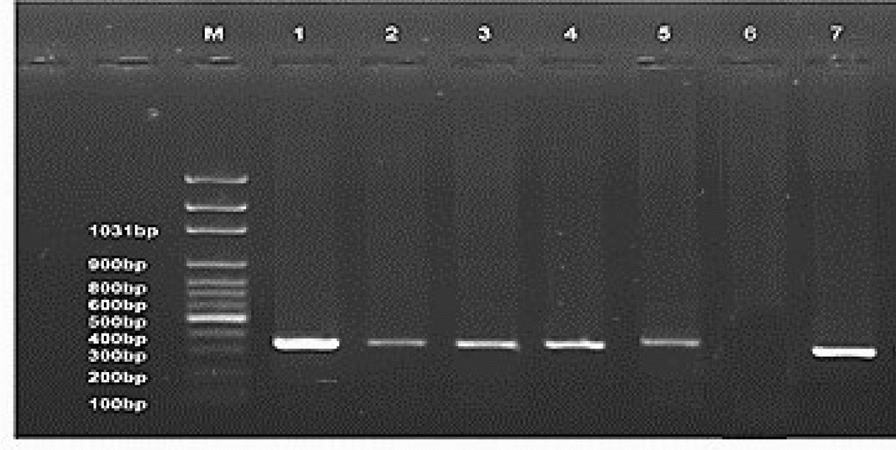
Identification of REV using PCR assay. PCR products at 370 bp of *LTR* gene of REV as lanes 1–5 are positive samples; lanes 6 is negative control; Lane 7 is positive control. M: represents a 100-bp ladder as a size standard

**Fig. 6:**
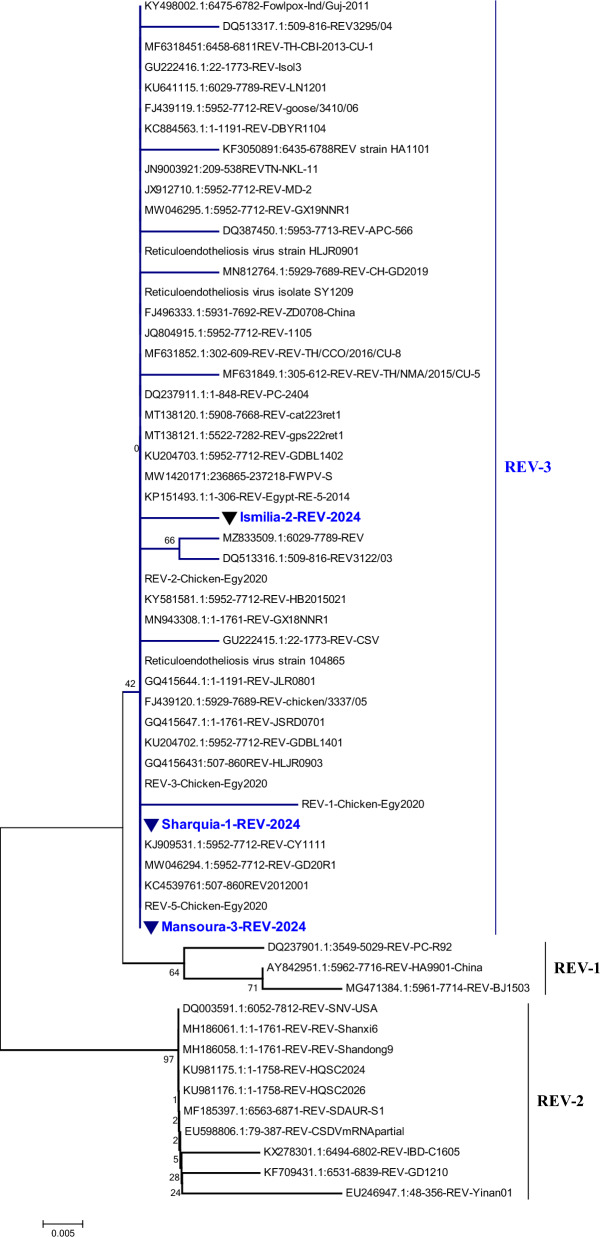
Collective phylogenetic tree based on *LTR* gene sequences alignment of REV comparable to other reference sequences in Genbank database. The phylogenetic analysis of the REV *LTR* gene showed that our REV three isolates located in subtype III (Sharquia-1-REV, Ismilia-2-REV, Mansoura-3-REV) with other Egyptian strains cluster in the same subtype. The REV three isolates in this study are identified by a triangle. The tree was constructed by the neighbor-joining method with 1,000 repeats bootstrap, using MEGA 7.0 software

A PCR-amplified product at 370 bp corresponding to a fragment of the targeted *LTR* gene was detected, which is similar to that of the positive control. Collectively, thirty tissue specimens were examined with only 5 samples were positive (16.6%) (Fig. 5, Table 3). Phylogenetic analysis was performed including 56 REV strains deposited in the Genbank database. Importantly, the phylogenetic tree construction corresponding to *LTR* gene fragment analysis (Fig. 6), revealed that all referred REV isolates (Sharquia-1-REV, Ismilia-2-REV, Mansoura-3-REV) were clustered into subtype III with accession numbers (PP763709, PP763710, PP763711); comparable to APC-566, CY111, MD-2, SY1209, chicken/3337/05, goose/3410/06, 104865-USA referential strains. Interestingly, Sharquia-1-REV has the highest genetically related to subtype III as APC-566 (American isolate), CY111, MD-2, SY1209 (Chinese isolates), chicken/3337/05, goose/3410/06 (Taiwanese isolates), and Egypt-RE-5-2014, REV-5-Chicken-Egy2020 (Egyptian isolates) with nucleotide identity percentage 100% as well as on the amino acid identity level were 99–100%; respectively (Table 4). Likewise, Ismilia-2-REV, Mansoura-3-REV have the greatest genetically correlated to APC-566, CY111, MD-2, SY1209, chicken/3337/05, goose/3410/06, Egypt-RE-5-2014, and REV-5-Chicken-Egy2020 with nucleotide identity percentage 99% and on the amino acid level were with (98–99%), (99–100%); respectively. Furthermore, Sharquia-1-REV isolate were close genetically similar to HA9901-China (subtype I), SNV-USA, REV-IBD-C1605-China (subtype II) isolates with nucleotide identity percentages of 99%, 97%, and 97%; respectively, and on the amino acid level were 98%, 96%, 95%; respectively. Similarly, Ismilia-2-REV isolate, and Mansoura-3-REV isolate shared nucleotide identity percentages of 98%, (97%, 96%), 96%; respectively, with HA9901-China, SNV-USA, REV-IBD-C1605-China, and based on the amino acid identity, the percentages were (97%, 98%), (95%, 96%), (94%, 95%); respectively (Table 4). Taken into account, amino acid sequencing analysis of the *LTR* gene of three isolates revealed 99% similarity to each other and 99% identity based on nucleotide identity level.

**Table 3:** REV detection using PCR method in various tissues of breeders

Tissue	Examined samples	Positive-PCR results	
		Number	Percentage
Liver	10	1	10%
Spleen	6	1	16.6%
Kidney	7	1	14.2%
Heart	3	0	0%
Bursa	1	1	100%
Ovaries	3	1	33.3%
Total number	30	5	16.6%

**Table 4:**
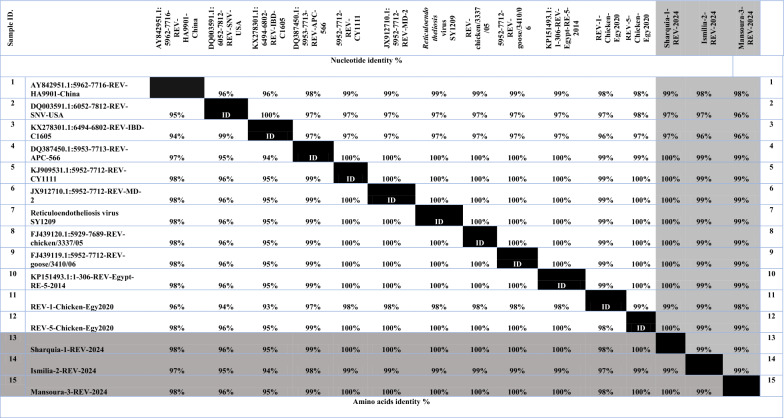
Nucleotide identities and divergence of sequenced virus isolates comparable to other selected strains

Nucleotide identities and divergence of our sequenced REV isolates comparable to other selected strains from China, Taiwan, Egypt, and the USA. The figure utilizes a comparative alignment of the *LTR* gene in which, the *LTR* nucleotide identity percentage of our three Egyptian isolates ranges from 96 to 100% comparable to other various referential strains. Besides, the amino acids identity percentage of our three Egyptian isolates ranges from 94 to 100% comparable to other various referential strains

## Discussion

Avian immunosuppressive retroviruses, referred to *Avian leucosis virus* and *Reticuloendotheliosis* virus, were a serious threat causing significant economic losses in the poultry industry globally [25–27]. Importantly, REV genes which integrate in the host genome causes immunosuppression as a result of atrophied immune organs which may lead to decrease the antibody levels achieved by vaccinations and can then increase the host susceptibility to secondary infections [28]. Also, it is mostly stated that the *LTR* gene can integrate specifically (permanent and stable insertion) into the genome of MDV and FWPV inducing a serious lymphoid organ atrophy in chickens [9–11]. Previous studies have explained that T lymphocytes were damaged in the thymus and spleen causing a decreased level of interleukin (IL)-2 secretion [28] resulting in a major immune dysfunction with alteration in the differentiation of T lymphocytes into T-helper cells and T-cytotoxic cells [29]. After REV infection, the expression levels were significantly down-regulated of differentially expressed genes [(ILs, tumor necrosis factor (TNFs), and interferons (IFNs)] in the infected cells. Replication of REV genome after the viral entry necessitate s an initial RNA reverse transcription step followed by an integration of the REV proviral DNA into the host cell genome through the integrase enzyme [30]. Notably REV eradication is very crucial for breeder flocks to overcome its rapid spreading. At present, REV is challenging to control as there is no available effective vaccination schedule. Therefore, the only accessible control method achieved through keeping flock renewal with the elimination of infected birds or cautiously selecting non-infected breeders [8, 31, 32]. Interestingly, REV infection were detected in Egypt in commercial chicken, turkey flocks [17, 33], ducks [34], and other avian species with high mortality rates [5]. The current data provides intensive interesting findings of REV screening in broiler breeder flocks in Egypt with subsequent confirmation through molecular characterization and sequencing analysis.

As depicted in our results, the positive breeder flocks with a seroprevalence rate of 35%, in El-Sharqia, El-Dakahliya, and Ismailia governorates have been shown clinical manifestation as weakness, growth retardation, face swelling, runting-stunting symptom, messy feathering, and leg lameness; attributing to depleting effect of lymphomas. Concerning post-mortem examinations among the field infected flocks, the liver, spleen, and kidneys are the most affected organs. More importantly, the necropsy findings included emaciated or stunted carcasses, congested, and uniformly enlarged liver, spleen, and kidney with grayish-white nodular infiltrations in investigated birds. The abdominal enlargement, with diffuse minute tumor foci which give a mottled or granular appearance in some cases, was due to the greatly enlarged liver and spleen. These results were in line with the findings of [5, 8, 17, 27]. Subsequently, REV also has immunosuppressive syndromes [35] so, mixed bacterial infection especially *E. Coli* species [36] and *Salmonella* species [37] may occur; causing increased mortalities, a drop in egg production, and economic losses associated with reduced efficiency. Moreover, the recorded mortality rate was 1.5–5%, whereas the morbidity rate was 3%. In addition, these findings are nearly in agreement with [5] who reported that the mortality rate of affected flocks ranged from 3 to 10%; correlating with secondary bacterial infection and farm management.

Pathological assessment of REV-induced lymphomas and other lesions presents difficulties [38]. In chicken flocks, REV can induce a lymphoma indistinguishable grossly and microscopically from *Avian leucosis virus* infection [25]. In this regard, histopathological evaluation of the examined organs concluded the presence of a lymphoreticular cell tumor. Also, the virus includes the *v-rel* gene, a viral oncogene, that is capable of acute reticulum cell neoplasia [35]. The focal or diffuse aggregations of lymphoblastic and reticular cells compress the adjacent cells; causing pressure atrophy. The same subsequent findings were recorded by [39, 40]. Additionally, the ovaries of most infected chickens were undeveloped; resulting in decreased egg production, and microscopically massive infiltrations of lymphoblast cells were observed. These subsequent results came in accordance with [5]. In the term of immunohistochemistry test [41], reported that immunohistochemistry was a potent diagnostic tool for REV detection as the microscopic lesions of REV infection could not be well differentiated from others caused by lymphoid leukosis and *Marek’s disease virus*. The immunohistochemistry technique has been routinely used to differentiate various avian oncogenic viruses [25, 42]. Notably, immunohistochemical staining revealed a moderate to strong reactivity for REV antigens in the cytoplasm as well as the nucleus of the infiltrating lymphoblastic cells in all examined organs except the heart. Hence, the heart didn't show any reactivity that may be attributed to low viral tropism. Previous reports have also described a positive reactivity of REV in both the cytoplasm and the nuclei of cells infected with REV [43]. [44, 45] reported a positive reactivity of REV in the liver, spleen, lungs, and kidneys collected from infected birds, as well as immunohistochemistry, considered a sensitive technique as compared to gross and microscopical diagnosis of various neoplasms caused by avian oncogenic viruses. [27, 46] concluded that the immunohistochemistry test was a differential diagnostic tool of REV in broiler breeders.

Regarding REV CPE, the CEF tissue culture showed aggregation, rounding, and massive detachments of cells. These results aligned with the CPE findings of [5, 21, 47, 48]. Additionally, [49] reported that the inoculated REV in CEF tissue culture needs an additional two or three passages to produce a reliable cytopathic effect. At the same time, the REV serological survey using a commercial ELISA test reported that only 7 breeder farms were considered positive with 35%. Likewise, only 70 serum samples were positive for the REV antibody with seroprevalence rates of 35%. The age of the tested flocks ranged from 23- 61 weeks to elude the false positive results derived passively from maternal-derived antibodies which can interfere with the REV seroprevalence under field conditions. Collectively, these current findings indicated the continuous distribution of REV in the examined breeder flocks, in Egypt. Interestingly, REV distribution in these breeder flocks may be attributed to spread vertically, causing an infection with REV and impairment of immune organs in younger chicks, resulting in diminished host immune response. These serology findings agree practically with previous studies conducted by [39], who mentioned a serological prevalence for the REV antibody of 25–100% at the 12th, and 25th weeks of cross-breed chickens in Delta Egypt. Also in China, our results came nearly in accordance with [50] who reported REV seropositive rates in native chicken flocks with a percentage of 32.2%. Besides, our observations also align with those findings documented in layer chicken in Taiwan by [51]. In contrast, our subsequent results are not in agreement with [34], who stated that REV antibody seroprevalence reached 66.7% of serum samples in four broiler breeders out of six, in Egypt. Additionally, [52] investigated that the seroprevalence of REV in Sudan was 69.2% for local breeds and 79.5% for commercial breeds of chickens; attributing to contaminated vaccines in commercial chickens. Concerning the aforementioned data, this variation in prevalence between countries may be due to the various strains and sample size, test conditions, types of chicken, environmental differences between geographical locations, transmission vertically, and contamination in poultry vaccines.

The most accurate and rapid method of choice for the REV diagnosis is PCR, which also supplies epidemiological data of REV isolates periodically. Particularly, PCR test with REV-specific primers confirmed only 5 samples are considered positive PCR samples with a percentage of 16.6%. These present molecular results are similar to those [52] who recorded the prevalence of positive PCR samples were 15% for the spleen and 10% for the liver; respectively. Parallel to our results, [53] assessed an approximately similar prevalence in layers in India. Furthermore, our findings align with [33] who indicated the positive PCR samples with REV with a percentage of 16.7% in breeder flocks. Nevertheless, our results were distinctly apparent from [5] who mentioned positive PCR results for REV with a percentage of 39.23% in chickens, in Thailand. Additionally, [2] initially explored REV infection presence at a high frequency in the tested samples with 65%. Concerning our results, [33] suggested that the Lower Egypt region farms in El Sharqia, El-Monofia, EL Daqahylia, EL Gharbia, EL Qalyoubia, and El Beheira governorates have an evidence of REV infection based on PCR assay in various chicken samples. Notably, our REV three isolates corresponding to the *LTR* gene were partially sequenced, systematically analyzed, and then compared with other referential sequences. According to the genetic characteristics, our REV isolates were clustered as subtype III; which is the most common subtype of REV circulating in a variety of avian species globally [9]. In the same line, the phylogenetic analysis revealed that the isolates of Sharquia-1-REV, Ismilia-2-REV, and Mansoura-3-REV were genetically correlated to American REV reference strains, Chinese REV reference strains, Taiwanese REV reference strains, and Egyptian strains with nucleotide similarity percentage 100%, 99%, 99%; respectively, as well as the amino acid identity percentage were (99–100%), (98%, 99%), (99%, 100%); respectively. Our findings are consistent with [2] who reported that the nucleotide identity of USP-586 and USP-976 strains ranged from 99.7 to 99.9% against APC-566 and 104,865 reference strains. Meanwhile, [5, 8, 33, 48] indicated a high percentage of identity in nucleotides and amino acids levels of their isolates with the strains of REV subtype III in China, Taiwan, Thailand, and the USA based on *env* gene analysis. In Egypt, REV isolates of *LTR* sequences showed a high degree of genetic identity to other REV-*LTR* integrated in Fowlpox virus (field-isolated and vaccine strains), Turkeypox virus isolates, Canarypox virus isolates [54], and MDV isolates [17]. However, we cannot detect the exact origin of our REV strains here, since there are two transmission routes of REV as well as through the contaminated vaccines. In accordance with the sequence analysis, our three REV isolates showed high homology to HA9901strain (subtype I), SNV, and REV-IBD-C1605 strains (subtype II) with nucleotide identity percentages ranging from 96 to 99%. Similar results were reported by [2] who stated a nucleotide similarity of REV isolates of 98.1% against REV subtype 1, and 95.2–96.5% against REV subtype 2. Also, our subsequent results came in accordance with [5] who indicated a nucleotide similarity of REV isolates of 95.8–96.1% with subtypes I and II (170A and SNV). Surprisingly, our three referred REV isolates based on the *LTR* gene showed 99% nucleotide similarity to each other, although the samples were collected from various regions and times. These findings demonstrated the low genetic variation of REV strains circulating in chicken flocks, in Egypt; which was consistent with numerous previous studies [2, 5, 8, 9, 33, 55].

## Conclusions

In conclusion, our current data collectively demonstrated the presence and molecular characteristics of REV strains circulating in broiler breeder flocks at various localities of Egypt through tissue culture, serological assays, immunopathological examination, and different genetic approaches. REV causes neoplastic and immunosuppressive manifestation with a seroprevalence rate of 35% and has been continuously distributed in three high poultry production Egyptian governorates. Moreover, the REV-*LTR* gene analysis of the isolates of Sharquia-1-REV, Ismilia-2-REV, and Mansoura-3-REV is highly similar to American, Chinese, Taiwanese REV reference strains, and also Egyptian strains with nucleotide identity percentage 100%, 99%, 99%; respectively, as well as the amino acid identity level were (99–100%), (98%, 99%), (99%, 100%); respectively. Since there are no available vaccinations or medications for REV, so REV still become a great harm to the local industry. Our study raises the awareness of REV as the causative agent of avian oncogenic disease in Egypt. Thus, the only accessible control and prevention method achieved through keeps flock renewal with the elimination of positive breeders, and implementing continuing genetic monitoring for all circulating strains. Additionally, in order to prevent or manage REV outbreaks, a full genome sequencing of these current isolates is advised in order to identify the pathogenicity, antigenic and genetic characteristics of the circulating strains in chicken populations.

## Supplementary Information

Supplementary material 1

Supplementary material 2

## Data Availability

No datasets were generated or analysed during the current study.
