# Supplementary material for: Molecular characterization and immunopathological investigation of Avian reticuloendotheliosis virus in breeder flocks in Egypt
Source: Virol J. 2024 Oct 22;21:259. doi: 10.1186/s12985-024-02525-5 (PMC11515750; doi:10.1186/s12985-024-02525-5)
Supplement: Supplementary file 1 — Supplementary material 1 [file 12985_2024_2525_MOESM1_ESM.docx]

| **Sample number** | ***S/P ratio of sera collected from diseased**  **breeder flocks** | **interpretation** | **Sample number** | **S/P ratio of diseased breeder flocks** | **interpretation** |
| --- | --- | --- | --- | --- | --- |
| **1** | 0.32 | Neg | **100** | 0.44 | Neg |
| **2** | 0.33 | Neg | **101** | 0.48 | Neg |
| **3** | 1.15 | Pos. | **102** | 0.32 | Neg |
| **4** | 0.26 | Neg | **103** | 0.34 | Neg |
| **5** | 0.65 | Pos. | **104** | 0.30 | Neg |
| **6** | 0.12 | Neg | **105** | 0.37 | Neg |
| **7** | 0.83 | Pos. | **106** | 0.19 | Neg |
| **8** | 1.18 | Pos. | **107** | 0.45 | Neg |
| **9** | 0.24 | Neg | **108** | 1.31 | Pos. |
| **10** | 0.68 | Pos. | **109** | 1.25 | Pos. |
| **11** | 0.82 | Pos. | **110** | 0.42 | Neg |
| **12** | 0.40 | Neg | **111** | 0.30 | Neg |
| **13** | 0.34 | Neg | **112** | 0.35 | Neg |
| **14** | 0.46 | Neg | **113** | 0.32 | Neg |
| **15** | 0.24 | Neg | **114** | 0.96 | Pos. |
| **16** | 1.16 | Pos. | **115** | 0.76 | Pos. |
| **17** | 1.29 | Pos. | **116** | 0.42 | Neg |
| **18** | 0.18 | Neg | **117** | 0.43 | Neg |
| **19** | 0.35 | Neg | **118** | 0.38 | Neg |
| **20** | 1.35 | Pos. | **119** | 0.29 | Neg |
| **21** | 0.62 | Pos. | **120** | 0.88 | Pos. |
| **22** | 0.25 | Neg | **121** | 0.85 | Pos. |
| **23** | 1.20 | Pos. | **122** | 0.19 | Neg |
| **24** | 1.14 | Pos. | **123** | 0.42 | Neg |
| **25** | 0.49 | Neg | **124** | 0.30 | Neg |
| **26** | 0.84 | Pos. | **125** | 0.35 | Neg |
| **27** | 1.36 | Pos. | **126** | 0.41 | Neg |
| **28** | 1.45 | Pos. | **127** | 0.56 | Pos. |
| **29** | 0.19 | Neg | **128** | 0.89 | Pos. |
| **30** | 1.16 | Pos. | **129** | 0.43 | Neg |
| **31** | 1.27 | Pos. | **130** | 0.35 | Neg |
| **32** | 0.26 | Neg | **131** | 0.73 | Pos. |
| **33** | 0.31 | Neg | **132** | 0.42 | Neg |
| **34** | 0.20 | Neg | **133** | 0.43 | Neg |
| **35** | 0.46 | Neg | **134** | 0.41 | Neg |
| **36** | 1.16 | Pos. | **135** | 0.36 | Neg |
| **37** | 1.24 | Pos. | **136** | 0.34 | Neg |
| **38** | 0.59 | Pos. | **137** | 0.46 | Neg |
| **39** | 1.18 | Pos. | **138** | 0.33 | Neg |
| **40** | 0.14 | Neg | **139** | 0.40 | Neg |
| **41** | 1.25 | Pos. | **140** | 0.57 | Pos. |
| **42** | 0.35 | Neg | **141** | 0.45 | Neg |
| **43** | 0.42 | Neg | **142** | 0.36 | Neg |
| **44** | 1.23 | Pos. | **143** | 0.29 | Neg |
| **45** | 0.15 | Neg | **144** | 0.35 | Neg |
| **46** | 1.34 | Pos. | **145** | 0.62 | Pos. |
| **47** | 1.15 | Pos. | **146** | 0.54 | Pos. |
| **48** | 0.46 | Neg | **147** | 0.38 | Neg |
| **49** | 1.26 | Pos. | **148** | 0.15 | Neg |
| **50** | 0.36 | Neg | **149** | 0.34 | Neg |
| **51** | 0.46 | Neg | **150** | 0.42 | Neg |
| **52** | 0.35 | Neg | **151** | 0.40 | Neg |
| **53** | 0.66 | Pos. | **152** | 0.41 | Neg |
| **54** | 0.26 | Neg | **153** | 0.46 | Neg |
| **55** | 0.15 | Neg | **154** | 0.25 | Neg |
| **56** | 0.27 | Neg | **155** | 0.64 | Pos |
| **57** | 0.88 | Pos. | **156** | 0.29 | Neg |
| **58** | 0.37 | Neg | **157** | 0.38 | Neg |
| **59** | 0.18 | Neg | **158** | 0.25 | Neg |
| **60** | 0.65 | Pos. | **159** | 0.87 | Pos |
| **61** | 0.20 | Neg | **160** | 0.55 | Pos |
| **62** | 0.14 | Neg | **161** | 0.33 | Neg |
| **63** | 0.88 | Pos. | **162** | 0.14 | Neg |
| **64** | 0.39 | Neg | **163** | 0.36 | Neg |
| **65** | 0.28 | Neg | **164** | 0.44 | Neg |
| **66** | 0.32 | Neg | **165** | 0.56 | Pos |
| **67** | 0.75 | Pos. | **166** | 0.49 | Neg |
| **68** | 0.48 | Neg | **167** | 0.36 | Neg |
| **69** | 0.15 | Neg | **168** | 0.58 | Pos. |
| **70** | 1.36 | Pos. | **169** | 0.98 | Pos. |
| **71** | 0.64 | Pos. | **170** | 0.30 | Neg |
| **72** | 0.38 | Neg | **171** | 0.52 | Pos |
| **73** | 0.19 | Neg | **172** | 0.35 | Neg |
| **74** | 0.38 | Neg | **173** | 0.41 | Neg |
| **75** | 0.28 | Neg | **174** | 0.29 | Neg |
| **76** | 0.27 | Neg | **175** | 0.92 | Neg |
| **77** | 0.38 | Neg | **176** | 0.46 | Neg |
| **78** | 0.15 | Neg | **177** | 0.34 | Neg |
| **79** | 0.34 | Neg | **178** | 0.68 | Pos. |
| **80** | 0.22 | Neg | **179** | 0.92 | Pos. |
| **81** | 0.63 | Pos. | **180** | 1.50 | Pos |
| **82** | 0.55 | Pos. | **181** | 0.31 | Neg |
| **83** | 0.29 | Neg | **182** | 0.21 | Neg |
| **84** | 0.67 | Neg | **183** | 0.42 | Neg |
| **85** | 0.39 | Neg | **184** | 0.33 | Neg |
| **86** | 0.18 | Neg | **185** | 1.36 | Pos. |
| **87** | 0.45 | Neg | **186** | 0.55 | Pos. |
| **88** | 0.43 | Neg | **187** | 0.49 | Neg |
| **89** | 0.24 | Neg | **188** | 0.68 | Neg |
| **90** | 0.16 | Neg | **189** | 0.27 | Neg |
| **91** | 1.24 | Pos. | **190** | 0.29 | Neg |
| **92** | 0.89 | Pos. | **191** | 2.45 | Pos |
| **93** | 0.47 | Neg | **192** | 0.39 | Neg |
| **94** | 0.28 | Neg | **193** | 0.89 | Neg |
| **95** | 0.39 | Neg | **194** | 0.22 | Neg |
| **96** | 1.15 | Neg | **195** | 0.33 | Neg |
| **97** | 0.66 | Pos. | **196** | 0.49 | Neg |
| **98** | 0.49 | Neg. | **197** | 0.58 | Pos. |
| **99** | 1.63 | Pos. | **198** | 1.37 | Pos. |
|  |  |  | **199** | 0.44 | Neg |
|  |  |  | **200** | 0.35 | Neg |

| Item | Positive | Negative |
| --- | --- | --- |
| Mean | 0.96 | 0.35 |
| Standard Deviation | 0.30 | 0.14 |
