# Supplementary material for: Molecular characterization and immunopathological investigation of Avian reticuloendotheliosis virus in breeder flocks in Egypt
Source: Virol J. 2024 Oct 22;21:259. doi: 10.1186/s12985-024-02525-5 (PMC11515750; doi:10.1186/s12985-024-02525-5)
Supplement: Supplementary file 2 — Supplementary material 2 [file 12985_2024_2525_MOESM2_ESM.docx]

- To diagnose and analyze REV by cell cultures, enzyme-linked immunosorbent assay (ELISA), histopathological investigation, the polymerase chain reaction (PCR) test, and sequencing analysis, 200 blood samples, and 50 tissue specimens were collected. The current study targets the occurrence and genetic characteristics of a viral neoplastic disease, resembling REV infection, circulating in breeder flocks from 2022-2023 in the Ismailia, El-Sharqia, and El-Dakahliya governorates using various diagnostic methods.

- REV was isolated on chicken embryo fibroblast cell culture; exhibiting cell aggregation, rounding, and cell detachments. Collectively, only 70 serum samples were positive for anti‐REV antibodies with seroprevalence rates of 35% based on the ELISA test. The histopathological observation demonstrated lymphoreticular tumors in the liver, spleen, and other examined organs. The immunohistochemical staining method confirmed the REV-positive signals in all examined organs (liver, kidney, spleen, bursa, ovaries) except for the heart. The PCR assay of the *LTR* gene assessed 370 base pairs with only 5 positive samples with a percentage of 16.6%. Three positive samples were further sequenced and submitted to the Genbank under accession numbers (PP763709, PP763710, PP763711). Phylogenetic analysis of the REV-*LTR* gene showed that our three isolates (Sharquia-1-REV, Ismilia-2-REV, Mansoura-3-REV) are REV subtype III which predominantly circulated in breeders in Egypt. These three isolates are highest similar to American, Chinese, and Taiwanese REV reference strains, and other Egyptian strains with nucleotide identity percentages of 100%, 99%, and 99%; respectively, and on the amino acid identity level were with (99%-100%), (98%, 99%), (99%, 100%); respectively.

-
